# Willingness to Use a Mobile Application for Scheduling Consultation Appointments Among Patients, Caregivers, and Healthcare Providers in Southwestern Uganda

**DOI:** 10.7759/cureus.106097

**Published:** 2026-03-30

**Authors:** Edgar Mulogo, Nuriat Nambogo, Nura Izath, Martin Mukama, Emmanuel Kamuhire, Davis Ntamakemwa, Moses Openja, Data Santorino

**Affiliations:** 1 Department of Community Health, Mbarara University of Science and Technology, Mbarara, UGA; 2 Department of Computer Science, Centre for Affordable Medical Technologies (CAMTech), Mbarara University of Science and Technology, Mbarara, UGA; 3 Department of Applied Science and Technology, Mbarara University of Science and Technology, Mbarara, UGA; 4 Department of Computer Engineering, Mbarara University of Science and Technology, Mbarara, UGA; 5 Department of Pediatrics, Mbarara University of Science and Technology, Mbarara, UGA

**Keywords:** application, appointment, caregiver, consultation, healthcare provider, mobicare, mobile, patient, scheduling, uganda

## Abstract

Background

Mobile applications are increasingly used to support healthcare delivery in Uganda. MobiCare is a mobile application developed to facilitate the scheduling of patient appointments at health facilities. This study assessed the willingness of patients/caregivers and healthcare providers to use the MobiCare application in private health facilities in Mbarara Municipality, Uganda.

Methods

A cross-sectional survey was conducted between July and September 2019 in five private hospitals in Mbarara Municipality. Structured interviewer-administered questionnaires were used to collect data from 97 patients/caregivers and 32 healthcare providers. Descriptive statistics were used to summarize findings, and inferential analysis was conducted among healthcare providers.

Results

All patients/caregivers owned a smartphone and reported internet use (97/97; 100.0%), and nearly all had previously used a mobile application (96/97; 99.0%). Awareness of mobile applications linking patients to healthcare providers was low (8/97; 8.2%). Willingness to use MobiCare was high among patients/caregivers (97/97; 100.0%) and healthcare providers (31/32; 96.9%). Most patients/caregivers recommended the application for scheduling appointments for children under five years (92/97; 94.8%). Among healthcare providers, dissatisfaction with existing appointment scheduling systems was associated with willingness to use MobiCare (OR = 3.7, 95% CI: 1.2-11.4; p = 0.02).

Conclusion

Willingness to use the MobiCare application was high among patients/caregivers and healthcare providers in private health facilities in Mbarara Municipality. Widespread smartphone ownership and internet use, together with dissatisfaction with existing appointment systems among providers, suggest favorable conditions for introducing digital appointment-scheduling tools in urban private healthcare settings. Further implementation research is needed to assess real-world uptake, usability, and impact on service delivery.

## Introduction

Digital technologies have continued to transform healthcare systems globally, with mobile applications increasingly adopted to enhance patient engagement and service delivery. Recent estimates indicate that nearly two‑thirds of the global population used the internet in 2022 [1]. In low‑income countries, internet utilization continues to rise despite persistent coverage challenges [2]. Mobile health (mHealth) applications have been increasingly adopted to improve access to care, patient-provider communication, and service efficiency, particularly in low- and middle-income countries [3-7].

In Uganda, smartphone penetration has expanded due to increased broadband availability and declining service costs [8]. Appointment-scheduling applications have been shown to reduce waiting times, improve clinic organization, and enhance patient satisfaction in various health system contexts [9-13]. Mobile health interventions have been increasingly explored in sub-Saharan Africa as a means of addressing health system challenges such as long waiting times, inefficient appointment management, and limited patient-provider communication [14-16]. Growing smartphone penetration and mobile internet access in urban settings have further supported the feasibility of deploying mobile appointment-scheduling applications in private healthcare facilities [14-16].

Most patients in Uganda seek care without prior scheduling, leading to long queues, delays, and reliance on informal sources for medical advice. To address these gaps, the study team developed MobiCare, an Android‑based mobile application enabling patients to identify health facilities, choose a preferred provider, and request an appointment conveniently.

This study aimed to assess the willingness of patients/caregivers and healthcare providers to use the MobiCare mobile application for scheduling consultation appointments in private health facilities in Mbarara Municipality, Uganda. Findings were intended to inform the deployment and scaling of the application.

## Materials and methods

Study setting and sample size

A descriptive cross‑sectional survey was conducted between July and September 2019 in Mbarara Municipality, southwestern Uganda. The Municipality comprises six divisions with an estimated population of 195,013 residents. It has one public Regional Referral Hospital that has a 600-bed capacity and serves the southwestern region. There are also five general hospitals that are privately owned, and each has a 100-bed capacity. The Municipality and surrounding population are also served by a network of private medical centres and clinics, as well as public health centres. The sample size estimation and analytical approach followed standard methods for descriptive cross-sectional health research as described in the literature [17,18].

The sample size for patients/caregivers was calculated using the standard formula for descriptive cross‑sectional studies [19], assuming a 50% proportion (p = 0.5), 10% margin of error (d = 0.1), and 95% confidence interval. The resulting sample size was n = 96. A total of 97 patients/caregivers were ultimately interviewed. Patients and caregivers were recruited using probability proportionate to size sampling across the participating private hospitals. Within each facility, participants were consecutively approached and invited to participate until the required sample size was reached. Healthcare providers were purposively sampled from the participating facilities, with a maximum of six providers recruited from each facility to ensure representation of different cadres. Healthcare providers (n = 32) were purposively sampled (maximum six per facility). These were not included in the sample size calculation. Five private health facilities in the Municipality were visited.

Instruments, variables, and analysis

Two interviewer‑administered questionnaires (patient/caregiver and health worker versions) were used. The study questionnaires were developed by the research team based on the literature on mobile health adoption and appointment scheduling systems. The draft tools were reviewed by experts in digital health and public health research to assess content validity. Minor revisions were made to improve clarity and contextual relevance. The questionnaires were piloted among a small group of patients and healthcare providers from a facility not included in the final study sample to assess clarity, flow, and comprehension. Feedback from the pilot test was used to refine the wording and structure of several questions before final data collection. Full questionnaires are included in Appendices A and B. Key variables captured included socio‑demographics, health‑seeking behavior, smartphone ownership and digital literacy, and willingness to use MobiCare. As part of the interview, participants were shown a demonstration of the MobiCare app interface.

Data analysis

Data were analyzed using STATA version 13 (StataCorp LLC, College Station, TX, USA). Descriptive statistics were summarized using frequencies and percentages for categorical variables and medians with interquartile ranges for continuous variables. Associations between willingness to use MobiCare and participant characteristics were assessed among healthcare providers using Fisher’s exact test due to small expected cell counts. Variables with p < 0.20 at bivariate analysis and those considered relevant were entered into a multivariable logistic regression model. Variables considered in the logistic regression analysis included age, gender, professional cadre, years of work experience, and satisfaction with the existing appointment scheduling system. Statistical significance was set at p < 0.05. Among patients/caregivers, willingness to use MobiCare was universal (97/97; 100%); therefore, inferential tests for willingness were not performed due to the absence of outcome variability.

## Results

A total of 97 patients and 32 healthcare providers were interviewed during the survey.

Patient socio-demographic characteristics

Patient/caregiver background characteristics are shown in Table 1.

**Table 1:** Sociodemographic Characteristics of Patients/Caregivers (N = 97) Percentages are based on total respondents (N = 97). Values are presented as n (%) unless otherwise indicated.

Characteristic	Value
Gender - Female	59 (61%)
Gender - Male	38 (39%)
Age (years), Mean (SD)	30 (+8.8)
Education level - None	2 (2%)
Education level - Primary	10 (10%)
Education level - Secondary	19 (19%)
Education level - Tertiary	66 (69%)
Marital status - Single	35 (36%)
Marital status - Married	62 (64%)
Household monthly income (USD), Median	130
Respondent category - Caregiver	34 (56%)
Respondent category - Patient	43 (44%)

The mean age of patients/caregivers was 30.0 years (SD ±8.8). Most respondents were female (59/97; 60.8%), had attained tertiary education (66/97; 68.0%), were married (62/97; 63.9%), and were caregivers (54/97; 55.7%). The median household monthly income was USD 130.

The profiles of the health providers are shown in Table 2.

**Table 2:** Profile of Healthcare Providers (N = 32) Values are presented as n (%) unless otherwise indicated.

Charaterictics	Value
Gender - Female	10 (31%)
Gender - Male	22 (69%)
Age (years), Mean (SD)	31.1 (+6.2)
Cadre - Medical officer	7 (21.9%)
Cadre - Specialist: Obstetrics and Gynecology	3 (9.4%)
Cadre - Specialist: Physician	2 (6.3%)
Cadre - Specialist: Other	8 (25.0%)
Cadre - Other clinical cadres	12 (37.5%)
Years of work experience, Mean (SD)	1.0 (+1.1)

A total of 32 healthcare providers participated in the study. The mean age was 31.1 years (SD ±6.2), and most were male (22/32; 68.8%). Medical officers constituted 7/32 (21.9%) of respondents, while specialists accounted for 13/32 (40.6%), including obstetricians/gynecologists, physicians, and other specialties. The mean duration of work experience was 1.0 year (SD ±1.1).

Health-seeking behavior among the respondents

Table 3 shows the health-seeking behavior among the patients/caregivers.

**Table 3:** Health-Seeking Behavior of Patients/Caregivers (N = 97) Values are presented as n (%) unless otherwise indicated. IQR: interquartile range

Parameter	Value
Distance to health facility (km), Median (IQR; Range)	2.5 (7-9; 1-80)
Number of times care sought from the health facility in the last 12 months, Median (IQR; Range)	4 (1-11; 1-12)
Time care was most frequently sought - Morning	68 (70%)
Time care was most frequently sought - Afternoon	19 (20%)
Time care was most frequently sought - Evening	9 (9%)
Time care was most frequently sought - Night	1 (1%)
Ever been in a queue at a private health facility - No	16 (16%)
Ever been in a queue at a private health facility - Yes	81 (84%)
Waiting time after arrival at health facility (minutes), Median (IQR; Range)	120 (not recorded; 0-720)
Satisfied with waiting time (n = 84) - No	63 (75%)
Satisfied with waiting time (n = 84) - Yes	21 (25%)
Ever attended a private health facility on a scheduled appointment - No	35 (36%)
Ever attended a private health facility on a scheduled appointment - Yes	62 (64%)
Method used to schedule appointment among those with scheduled appointments (n = 62) - Phone call	43 (69%)
Method used to schedule appointment among those with scheduled appointments (n = 62) - Text/WhatsApp	4 (7%)
Method used to schedule appointment among those with scheduled appointments (n = 62) - Scheduled on previous visit	15 (24%)
Method used to schedule appointment among those with scheduled appointments (n = 62) - Mobile health application	0 (100%)
Method of scheduling worked well for patient (as recorded) - No	4 (6.3%)
Method of scheduling worked well for patient (as recorded) - Yes	60 (94%)

Participants reported a median waiting time of 120 minutes (range: 0-720). Among those who responded to the satisfaction question (n = 84), 63 (75.0%) were dissatisfied with the waiting time.

Phone ownership and use among the respondents

Table 4 shows the phone ownership and use characteristics among the patients/caregivers.

**Table 4:** Phone Ownership and Use Among Patients/Caregivers (N = 97) Percentages are calculated based on the total number of patients/caregivers (N = 97). Values are presented as n (%) unless otherwise indicated.

Parameter	Value
Own a mobile phone - Yes	97 (100%)
Own a mobile phone - No	0 (0.0%)
Own a smartphone - Yes	97 (100)
Own a smartphone - No	0 (0.0%)
Use the internet - Yes	97 (100)
Use the internet - No	0 (0.0%)
Ever used a mobile application - Yes	96 (99)
Ever used a mobile application - No	1 (1.0%)
Heard of an app linking patients to providers - Yes	8 (8.2%)
Heard of an app linking patients to providers - No	89 (91.8%)

All patients/caregivers reported owning a mobile phone (97/97; 100.0%), all of which were smartphones (97/97; 100.0%), and reported internet use (97/97; 100.0%). Nearly all respondents had previously used a mobile application (96/97; 99.0%). However, awareness of mobile applications specifically designed to link patients to healthcare providers was low, with only 8/97 (8.2%) reporting prior awareness of such applications.

Willingness to use the MobiCare app among the patients/caregivers

Table 5 presents the willingness to use MobiCare.

**Table 5:** Willingness to Use MobiCare Values are presented as n (%) unless otherwise indicated.

Parameter	Patients/Caregivers (N = 97)	Healthcare Providers (N = 32)
Willing to use MobiCare - Yes	97 (100.0%)	31 (96.9%)
Willing to use MobiCare - No	0 (0.0%)	1 (3.1%)
Who should use MobiCare (multiple responses allowed) - Pregnant women	81 (83.5%)	15 (46.9%)
Who should use MobiCare (multiple responses allowed) - Chronic illness patients	70 (72.2%)	0 (0.0%)
Who should use MobiCare (multiple responses allowed) - Caretakers of children under five years	92 (94.8%)	9 (28.1%)
Who should use MobiCare (multiple responses allowed) - Working class	85 (87.6%)	14 (43.8%)
Who should use MobiCare (multiple responses allowed) - Everyone	39 (40.2%)	18 (56.3%)
Would recommend MobiCare to others - Yes	95 (99%)	32 (100%)
Would recommend MobiCare to others - No	0 (0.0%)	0 (0.0%)

All patients/caregivers (97/97; 100.0%) expressed willingness to use MobiCare, compared with 31/32 (96.9%) healthcare providers. Among patients/caregivers, the application was most commonly recommended for caretakers of children under five years (92/97; 94.8%), while providers most frequently recommended its use for everyone (18/32; 56.3%).

Factors associated with willingness to use MobiCare (healthcare providers)

Table 6 presents a logistic regression analysis of factors associated with willingness to use MobiCare among healthcare providers.

**Table 6:** Logistic Regression Analysis of Factors Associated With Willingness to Use MobiCare Among Healthcare Providers (N = 32)

Variable	Odds Ratio (OR)	95% Confidence Interval	p-value
Dissatisfaction with the appointment system	3.7	1.2-11.4	0.02

Inferential analysis was conducted among healthcare providers because the willingness to use MobiCare among patients/caregivers was universal (97/97; 100.0%), and therefore lacked outcome variability required for statistical testing. In the bivariate analysis, dissatisfaction with existing appointment scheduling systems was significantly associated with willingness to use MobiCare. Healthcare providers who reported dissatisfaction with the current appointment system were more likely to express willingness to adopt the application compared with those who reported satisfaction (Fisher’s exact test p = 0.02).

Variables considered clinically relevant were subsequently included in a multivariable logistic regression model to identify predictors of willingness to use MobiCare among healthcare providers. In the adjusted model, dissatisfaction with the current appointment system remained significantly associated with willingness to use the application (OR = 3.7, 95% CI: 1.2-11.4; p = 0.02). No other provider characteristics, including age, gender, or professional cadre, were significantly associated with willingness to use MobiCare in the adjusted model (p > 0.05).

## Discussion

This study assessed the willingness of patients/caregivers and healthcare providers to use MobiCare, a mobile application designed to facilitate appointment scheduling in private health facilities in southwestern Uganda. The findings demonstrate a high level of willingness to use the application among both groups, supported by widespread smartphone ownership, internet use, and prior experience with mobile applications. These results suggest that the foundational digital prerequisites for adoption of mobile appointment-scheduling tools are present in this urban, private-sector setting. The study design, sample size estimation, and analytical approach followed standard methods for descriptive cross-sectional health research as described in the literature [17,18]. High willingness to adopt mobile health interventions has also been reported in other studies conducted in similar settings, particularly where existing appointment and patient-flow systems are perceived as inefficient [19]. This has also been reported elsewhere on the use of mobile health apps among caregivers [20]. It has, however, been reported that adults are reluctant to engage with new apps [21]. More importantly, however, the opportunity to schedule an appointment without having to go to the health facility and wait in line is likely to have influenced the willingness to use MobiCare. Among patients and caregivers, willingness to use MobiCare was universal, despite limited prior awareness of mobile applications specifically designed to link patients with healthcare providers. This indicates that willingness may be driven more by perceived need and anticipated benefits than by previous exposure to similar technologies. Long waiting times, frequent queuing, and dissatisfaction with existing appointment systems - documented in this study - likely contribute to receptiveness toward digital alternatives. The high proportion of respondents recommending MobiCare for specific population groups, particularly caretakers of children under five years and pregnant women, highlights the perceived utility of appointment scheduling for populations requiring frequent or time-sensitive healthcare visits. This is consistent with patterns elsewhere, where mobile phone applications run locally on smartphones, offering the possibility for permitting appointments to be booked and scheduled beyond standard operating hours [22,23]. The preference for such groups reflects both the burden of repeated facility visits and the potential for appointment scheduling to reduce waiting times and missed consultations.

Healthcare providers also demonstrated high willingness to use MobiCare, with inferential analysis indicating that dissatisfaction with existing appointment systems was significantly associated with willingness to adopt the application. Providers who were dissatisfied with current scheduling processes had higher odds of willingness to use MobiCare, underscoring the importance of addressing workflow inefficiencies in driving provider adoption of digital health tools. This contrasts with a study that found healthcare providers, although willing to discuss health apps, would not recommend them readily to healthcare seekers [24,25].

The dominance of phone calls, text messaging, and social media platforms such as WhatsApp as current appointment-scheduling methods, as reported by providers, further emphasizes the absence of standardized, systematized scheduling tools in this context. While these informal methods facilitate communication, they may be inefficient, inconsistently documented, and difficult to scale [26]. The limited prior use of dedicated mobile health applications for appointment scheduling suggests an unmet opportunity for structured digital solutions such as MobiCare.

Overall, the findings suggest that mobile appointment-scheduling applications such as MobiCare have strong potential to improve appointment management in private health facilities in Uganda. However, successful implementation will depend on usability, integration into existing facility workflows, data privacy safeguards, and sustained engagement from both patients and providers. Future research should focus on pilot implementation studies to evaluate actual utilization, user experience, and effects on waiting times, clinic efficiency, and patient satisfaction.

Study limitations

This study has a number of limitations. The sample size of healthcare providers was relatively small (n = 32), potentially constraining the statistical power of the inferential analyses. Second, the study took place only in private hospitals in a city. Consequently, the results may not be applicable to rural settings or public health institutions where digital accessibility and health system dynamics may vary. Third, the patients and caregivers who said they were very willing to use MobiCare may have been influenced by social desirability bias or excitement from seeing the app in action during the interview. Lastly, the study looked at stated willingness instead of actual use of the app, so it doesn't show how well the app is being used or adopted in the real world.

## Conclusions

This study found that both patients/caregivers and healthcare providers were very willing to use the MobiCare app to make appointments for consultations in private health facilities in Mbarara Municipality. These results indicate possible viability for digital appointment scheduling tools in analogous urban private healthcare environments. But being willing to do something doesn't always mean you'll actually do it. More studies on implementation are needed to find out how these applications work in the real world, how easy they are to use, and how they affect the delivery of health services.

## Appendices

Appendix A

Health Worker Pre-study for MobiCare Willingness Questionnaire

Questionnaire number………………………………………………….

Name of Interviewer…………………………………………………...

Date (DD/MM/YY) ……………………………………………………

Name of Health facility…………………………………………….......

A. Health Worker’s General information

A.0 Age…………………………………………………………………

A.1 Gender (M/F)……………………………………………………….

A.2 Specialization e.g. Pediatrician……………………………………..

A.3 Years of Experience

a). 1- to - 3 years   (b). 4- to - 6 years   (c). 7 - to-  9 years        (d). 10 years and above    

A.4 Registered

 Yes                              No           

B. Phone ownership and use

B.0 Do you currently own a smartphone(s)?

    Yes                         No

B.1 If Yes, do you use internet?

   Yes                         No      

B.2 Has a patient ever asked you for an appointment?

    Yes                        No

B.3 If yes, how did they ask for the appointment? (Tick all that apply)

   (a). Phone call               (b) text messages          (c). Social media (WhatsApp)                           (d). Mobile health application        (e) Other………………………………………. (Specify) 

B.4 Please tell us about your experience using that method (s) to schedule an appointment for the patient(s)?

…………………………………………………………………………………………………..

…………………………………………………………………………………………………..

…………………………………………………………………………………………………..

B. 5 If you were given an opportunity to design an appointment scheduling system, describe how the scheduling system would work? ………………………………………………………………

……………………………………………………………………………………………………...

……………………………………………………………………………………………………...

B.6 Have you ever used any mobile application to schedule appointments for your patients?

      Yes                       No

B.7 If yes, specify? …………………………………………………………………………………

………………………………………………………………………………………………………

B.8 How does it work?   ....................................................................................................................

………………………………………………………………………………………………………

………………………………………………………………………………………………………

 C.0 Application Acceptability

“Now I would like you to tryout our mobile health application called ‘MobiCare’, that is intended to link patients to health workers through a convenient appointment scheduling system.

********************************

Interviewer lets the health worker to tryout the application.

********************************

“Here are some questions on what you think about MobiCare”

C.1 What specific features do you like about MobiCare application?

 …………………………………………………………………………………………………

………………………………………………………………………………………………….

C.2 What specific features don’t you like about MobiCare application?

…………………………………………………………………………………………………

…………………………………………………………………………………………………

C.3 How in your opinion do you think MobiCare will be of help in making it easier to schedule appointments? …………………………………………………………………………..

…………………………………………………………………………………………………….

C.4 How will MobiCare influence the way you provide health care in a private health facility?

…………………………………………………………………………………………………….     

…………………………………………………………………………………………………….

C.5 Would you be willing to use the MobiCare in future?

      Yes               No

C.6 Why or Why not?

………………………………………………………………………………………………….

………………………………………………………………………………………………….

C.7 Who do you think should use the MobiCare application? (Tick all that apply)

       (a). Pregnant women     (b). Patients with chronic conditions (Diabetes, Hypertension, etc.)

       (c). Mothers/Caretakers of under-five children          (d). Working class       (e). Everyone             (f). Don’t know   (g). Other……………………………………………………………. (Specify)

C.8 Why…………………………………………………………………........................................

……………………………………………………………………………………………………..

C.9 What group of people should not use the MobiCare application?

 ……….……………………………………………………………………………………………

……………………………………………………………………………………………………..

C.10 Why…………………………………………………………………....................................

…………………………………………………………………………………………………….

C.11 What features would you recommend to be added in MobiCare? ………………………......

………………………………………………………………………………………………………

………………………………………………………………………………………………………

C.12 Would you recommend MobiCare to other people?

Yes               No

C.13 Why or Why not? …………………………………………………………………………..

……………………………………………………………………………………………………..

 “If the health worker has expressed willingness to use the MobiCare application, he or she will provide contact information”.      

                                        THANK YOU FOR YOUR TIME

Appendix B

Pre-study MobiCare Patient/Caregiver Willingness Questionnaire

Questionnaire number…………………………………………………………………..

Name of Interviewer…………………………………………………………………….

Date   (DD/MM/YY)…………………………………………………………………….                             

Name of Health Facility…………………………………………………………………

A. Participant’s General Information

A.0 District………………………………………………….

A.1 Residence……………………………………………….

A.2 Gender (M/F)……………………………………………         

A.3 Age……………………………………………………...

A.4 Date of birth…………………………………………….

A.5 Occupation……………………………………………..

A.6 What is your average monthly income in UGX?   

………………………………………………………………………………………………………     

A.7 Highest Level of Education

       (a). Never attended school                  (b). Primary                     (c). O’ Level        

        (d). A’ Level                                      (e). University                  (f). Other tertiary institutions

A.8 Now I would like you to read the sentence below

       I always come to this private health facility to consult about my health.

A.9 Marital status

       (a). Married              (b). Single                 (c). Windowed                        (d). Divorced

A.10 Are you here as a patient or caretaker?

         (a). As a patient      (b). As a caretaker

A.11 What is the average distance from your home to this health facility? .......................... (Kms)

A.12 How many times have you sought health care in a private health facility within the last twelve months? …………………………………………………………………………………

A.13 At what times do you most frequently seek health care in a private health facility?

         (a). Morning                      (b). Afternoon                 (c). Evening                (d). Night

A.14 Why at this time? ………………………………………………………………………….

          ……………………………………………………………………………………………..

A.15 Have you ever been in a private health facility queue to see a health worker?

       Yes                                                    No

A.16 Tell us about your experience in the queue? ………………………………………………..

       …………………………………………………………………………………………………

A.17 How long did it take you to consult the health worker from the time you arrived at the health facility? ............................................................................................. (Minutes)

A.18 Has this time worked for you?

Yes                            No

Why or why not? ………………………………………………………………………………..

B. Phone ownership and use

B.0 Do you currently own a phone/s?

    Yes                         No

B.1 Is it a smartphone?

    Yes                         No    

B.2 If Yes, do you use internet?

   Yes                         No   

B.3 Have you ever used a mobile application like whatsApp, Facebook, on your smartphone?

   Yes                         No    

B.4 Have you ever been to a private health facility on scheduled appointment?

    Yes                        No

B.5 If yes, how was the appointment scheduled? (Tick all that apply)

(a). Phone call              (b). Text/WhatsApp message           (c). Scheduled on previous visit     (d). Mobile health application  (e) other…………………… (Specify) (h) No one

B.6 Has this system of scheduling appointments worked for you?

   Yes                          No

B.7 Please tell us about your experience using that method of scheduling appointment?

…………………………………………………………………………………………………..

…………………………………………………………………………………………………..

B. 8 If you were given an opportunity to design an appointment scheduling system, describe how you would want it to work? ………………………………………………………………

……………………………………………………………………………………………………...

……………………………………………………………………………………………………...

B.9 Have you heard of mobile application/s that links patients to health workers for appointment scheduling?

      Yes                       No

B.10 If yes, specify? ………………………………………………………………………………

B.11 How does it work/s?   ..............................................................................................................

………………………………………………………………………………………………………

………………………………………………………………………………………………………

C.0 Application Acceptability

“Now I would like to tryout our mobile health application called ‘MobiCare’, that is intended to link patients to health workers through a convenient appointment scheduling system.

********************************

Interviewer lets the participant to tryout the application.

********************************

“Here are some questions on what you think about MobiCare”

C.1 What specific features do you like about MobiCare application?

 …………………………………………………………………………………………………

………………………………………………………………………………………………….

C.2 What specific features don’t you like about MobiCare application?

…………………………………………………………………………………………………

…………………………………………………………………………………………………

C.3 How in your opinion do you think MobiCare will be of help? ………………………………

…………………………………………………………………………………………………….

C.4 How will MobiCare influence the way you seek care in a private health facilities?

…………………………………………………………………………………………………….     

…………………………………………………………………………………………………….

C.5 Would you be willing to use the MobiCare in future?

      Yes               No

C.6 Why or Why not ?

………………………………………………………………………………………………….

………………………………………………………………………………………………….

C.7 Who do you think should use the MobiCare application?

       (a). Pregnant women     (b). Patients with chronic conditions (Diabetes, Hypertension, etc.)

       (c). Mothers/Caretakers of under-five children          (d). Working class       (e). Everyone             (f). Don’t know   (g). Other……………………………………………………………. (Specify)

C.8 What group of people should not use the MobiCare application?

 ……….……………………………………………………………………………………………

……………………………………………………………………………………………………..

C.9 What features would you recommend to be added in MobiCare? ………………………......

………………………………………………………………………………………………………

………………………………………………………………………………………………………

C.10 Would you recommend MobiCare to other people?

Yes               No

C.11 Why or Why not? …………………………………………………………………………..

……………………………………………………………………………………………………..

 If the patient/caretaker has expressed willingness to use the MobiCare application, he or she will provide contact information.      
